# A nebulized complex traditional Chinese medicine inhibits Histamine and IL-4 production by ovalbumin in guinea pigs and can stabilize mast cells *in vitro*

**DOI:** 10.1186/1472-6882-13-174

**Published:** 2013-07-13

**Authors:** Hung-Chou Chang, Cheng-Chung Gong, Chi-Lim Chan, Oi-Tong Mak

**Affiliations:** 1 Department of Life Sciences, National Cheng Kung University, Tainan, Taiwan, ROC; 2 Department of Medical Technology, Chung Hwa University of Medicine Technology, Jen-Te, Tainan, Taiwan, ROC; 3 Office of Academic Affairs, Tainan Tzu-Chi Senior High School, Tainan, Taiwan, ROC; 4 Department of Healthcare Management, University of Kang Ning, 188, Sec. 5, An Chung Rd. A Nan District, Tainan, Taiwan, ROC

**Keywords:** Asthma, Nebulization, Drug delivery, Chinese medicine, Ovalbumin

## Abstract

**Background:**

Traditional Chinese medicines have been used for anti-asthma treatment for several centuries in many Asian countries, and have been shown to effectively relieve symptoms. Our previous study demonstrated that a complex traditional Chinese medicine (CTCM) administered in nebulized form through the intratracheal route is effective against early-phase air-flow obstruction and can inhibit IL-5 production in ovalbumin (OVA)-sensitized guinea pigs. However, the antiasthmatic mechanisms of CTCMs are still unclear.

**Methods:**

In this study, we examined the underlying mechanism of a CTCM that we used in our previous study in order to ascertain its function in the early-phase response to OVA challenge.

In each group, 10–12 unsensitized or OVA-sensitized guinea pigs were treated with nebulized CTCM before OVA challenge, and the airway responses of the animals to OVA were recorded. Bronchoalveolar lavage fluid (BALF) samples were collected 5 min after OVA challenge, and the histamine and IL-4 contents in the BALF were measured. P815 cells (a mouse mast cell line) were untreated or pretreated with CTCM or cromolyn sodium (a mast cell stabilizer), and incubated with Compound 48/80 (mast cell activator) for 9 hr. The levels of histamine and IL-4 released from the cells were quantified.

**Results:**

We found that the inhibition of bronchoconstriction by the CTCM was attenuated by pretreatment with propranolol, suggesting that the CTCM has a bronchodilator effect that is associated with beta-adrenergic receptor. Our results also showed that the CTCM inhibited histamine and IL-4 secretion in the OVA-induced airway hypersensitivity in guinea pigs at 5 min post-OVA challenge, and *in vitro* study revealed that the CTCM is able to stabilize mast cells.

**Conclusion:**

In conclusion, our results suggested that the CTCM is a kind of bronchodilator and also a mast cell stabilizer. Our findings provide useful information regarding the possible mechanism of the CTCM, and show its potential for application in the treatment of allergenic airway disease.

## Background

Asthma is a complex syndrome with multiple causes, which include biological and environmental factors. Airway inflammation plays a key role in the onset of allergic asthma. The inflammation response is characterized by dual responses (early- and late-phase responses to allergen inhalation), acute and transient airway hyperresponsiveness (AHR) and infiltration of inflammatory cells (mast cells, lymphocytes, and eosinophils) into the airways [1-6]. Currently, inhaled corticosteroids with or without long-acting β agonists are the mainstay of treatment for asthma. However, large proportions of patients are poorly-controlled with this type of management. Anti-leukotrienes are currently used in therapy, but are less effective than inhaled corticosteroids [7].

In chronic inflammatory disorder of the airways, many cell types are involved, including eosinophils, lymphocytes, macrophages, neutrophils and mast cells. Of these, eosinophils are the most characteristic cell type that has been shown to be correlated with the severity of asthma [8], as several mediators resulting from eosinophil activation may contribute to the contraction of the airway smooth muscles [9,10]. In addition, mast cells have long been considered to play an important role in the pathophysiology of asthma, as they release a variety of mediators, including bronchoconstrictors (i.e., histamine, cysteinyl-leukotrienes and prostaglandins D2) [11-13]. Mast cells are thought to be a major source of IL-4 and other pro-inflammatory cytokines [14], and the secretion of cytokines may act as a trigger for the induction of subsequent persistent production of IL-4 and IL-5 by lymphocytes [15,16]. Activation of mast cells contributes to early-phase asthmatic responses (EAR), which involve airway smooth muscle constriction, vascular leakage, increased mucus production, enhanced AHR and recruitment of inflammatory cells [17]. It is well-established that cross-linking of IgE Abs on mast cells by Ag triggers the release of mediators and cytokines, which cause immediate allergic reactions [15,18-20].

Using animal models, studies have shown that several formulas of complex traditional Chinese medicines (CTCMs) could inhibit the EAR after stimulation of antigens [21-27]. These Chinese medicines are administered orally, and the mechanisms of their effects remain unclear. In our previous study, we demonstrated that a CTCM administered in nebulized form through the intratracheal route was effective against early-phase air-flow obstruction and inhibited IL-5 in bronchoalveolar lavage fluid (BALF) in ovalbumin (OVA)-sensitized guinea pigs [28]. The mechanism of the effect on asthmatic responses in sensitized guinea pigs after treatment with a CTCM is still unclear. Whether CTCMs stabilize mast cells and play a role in the relaxation of airway smooth muscle or the inhibition of airway contraction needs to be investigated. In this study, we used Compound 48/80 to activate mast cells, and the stabilization effect of a CTCM on mast cells was investigated by measuring the release of histamine and IL-4 from mast cells. In addition, the levels of histamine and IL-4 in the BALF of OVA-sensitized guinea pigs that inhaled the CTCM were analyzed. We used both *in vivo* and *in vitro* models to investigate the mechanism underlying the therapeutic effect of the CTCM in asthma treatment.

## Methods

### Sensitization and challenge of animals

The protocol for the animal experiment was reviewed and approved by the Animal Care and Use Committee of National Cheng Kung University. Sensitization of guinea pigs was performed as per the method described in our previous study [28]. Briefly, a total of 62 specific pathogen-free Dunkin-Hartley male guinea pigs (400 ~ 600 g) were purchased from the National Laboratory Animal Center (Taipei, Taiwan), and the animals were actively IgE-sensitized to OVA (grade VI; Sigma-Aldrich, St. Louis, MO, USA). Briefly, on day one of sensitization, guinea pigs were sensitized by intraperitoneal injection of OVA (100 μg with 10 mg of aluminum hydroxide [Al(OH)_3_] gel in 0.5 ml of normal saline), and a booster sensitization (50 μg of OVA and 5 mg of Al(OH)_3_ gel in 0.25 ml normal saline) was performed on day 7. Then, the sensitized guinea pigs were exposed to an aerosol of 1% OVA (w/v) in normal saline for 20 sec on day 14 using a Pulmo-Aide compressor (model 5650D; DeVilbiss, Somerset, PA, USA). For bronchoalveolar lavage collection and measurement of airway responses to OVA, the animals were anesthetized with urethane (2 g/kg i.p.), intubated, and challenged with 1% OVA (w/v) for 20 sec with/without CTCM for 5 min by nebulization on day 21. Unsensitized guinea pigs were treated in the same way with normal saline. The detailed treatment was as shown in Additional file 1: Figure S1A.

### Preparation of the Chinese herbal formula

In general, the EAR reaches a peak at about 5–30 min after challenge and lasts for around 2 hours, and the EAR is followed by the late-phase asthmatic responses (LAR), which occur 4–12 h after challenge and may last for several hours or even few days [29]. In this study, a CTCM formula similar to the *xiao-qing-long-tang* (XQLT) formula that is used by traditional Chinese medical physicians for the treatment of asthmatic patients was used. The XQLT formula has been proven to suppress the EAR and LAR in sensitized guinea pigs via oral administration [22]. The formula includes eight Chinese herbs: Ephedrae herba (stem of *Ephedrae sinica* Stapf, 18.75 g), Paeoniae radix (root of *Paeoniae lactiflora* Pallas, 18.75 g), Glycyrrhizae radix (root and rhizome of *Glycyrrhiza uralensis* Fischer, 18.75 g), Cinnamonomi ramulus (cortex of *Cinnamomum cassia* Blume, 18.75 g), Asari herba cum radice (whole plant of *Asarum sieboldii* Miq, 18.75 g), bitter apricot seed (*Prunus armeniaca* (Magnoliaceae), 18.75 g), common perilla (leaves of *Perilla frutescens* (Labiatae), 18.75 g), and Ledebouriella root (*Saposhnikovia divaricate* (Apiaceae), 18.75 g). The herbs were purchased from a government-approved herbal company (Hong-Cheng Chinese Medicine, Tainan, Taiwan). All dried herbs were dipped into 800 ml distilled water for 30 min, then boiled and maintained at about 103°C for 15 min. The resulting decoction was concentrated to approximately 350 ml, and was then cooled and filtered through a 0.45-mm filter. The decoction was finally lyophilized to yield 21 g dried powder, which was stored at 4°C. The dried extract was dissolved in saline before use.

### Experimental design and administration

Pulmonary resistance (**R**_**L**_) was measured before treatment and challenge as the baseline control. Then, the **R**_**L**_ was recorded after challenge every 5 min for the first hour and every 30 min during the following 5 hours. Bronchoalveolar lavage was performed 6 hr after ovalbumin challenge. The guinea pigs were randomly divided into six groups:

(1) NSGP_S (n = 10): unsensitized guinea pigs treated with saline by nebulization;

(2) NSGP_M (*n* = 10): unsensitized guinea pigs treated with 0.06 g/ml CTCM for 5 min by nebulization;

(3) SGP_O (*n* = 10): sensitized guinea pigs challenged with OVA by nebulization;

(4) SGP_MO (*n* = 12): sensitized guinea pigs treated with 0.06 g/ml nebulized CTCM for 5 min, followed by OVA challenge by nebulization (at 5 min post-CTCM nebulization);

(5) SGP_AMO (*n* = 12): sensitized guinea pigs pre-treated with β-adrenoceptor antagonist propranolol (1 mg/kg *i.v*.) for 3 min before treating with nebulized 0.06 g/ml CTCM for 5 min, and finally challenged by OVA by nebulization (at 5 min post-CTCM);

(6) NSGP_O (*n* = 10): unsensitized guinea pigs challenged with OVA by nebulization.

### Measurement of airway responses to OVA challenge

On day 21, airway responses were measured according to the method described by Abraham et al. [30]. Briefly, guinea pigs were anesthetized with urethane (2 g/kg; i.p.). A balloon catheter placed through one nostril into the end of esophagus was used to measure pleural pressure; and a catheter placed into the trachea was used to measure lateral pressure (Additional file 1: Figure S1B). Transpulmonary pressure was the difference between lateral and pleural pressure. A pneumotachograph was connected to the endotracheal tube, by which the signals of flow and transpulmonary pressure were recorded on a computer. The pulmonary resistance (RL) calculated from the transpulmonary pressure and flow at the isovolumetric points.

### Histamine and IL-4 in bronchoalveolar lavage fluid (BALF)

Bronchoalveolar lavage fluid (BALF) was collected 5 min after challenge. The lung lavage was performed gently, using 3 ml of PBS (phosphate-buffered saline) at 37°C. The tracheal cannula was clamped, and the thorax was massaged for 60 s before the BALF was recovered. This process was repeated once. The recovered lavage samples were cooled on ice immediately and centrifuged at 150 × *g* for 10 min at 4°C. Approximately 4 ml of fluid were recovered from each animal, thus maintaining a roughly equal amount of BALF among each of the treatment groups. After centrifugation, the histamine and IL-4 contents in the BALF were measured using a histamine enzyme immunoassay kit (Cayman Chemical, Paris, France) and an IL-4 ELISA kit (BD Biosciences, NJ, USA), respectively.

### Effects of the CTCM on the inhibition of histamine and IL-4 release in a mast cell culture

P815 cells (mouse lymphoblast-like mastocytoma cell line; ATCC #TIB-64) were maintained in DMEM medium supplemented with 10% fetal bovine serum, 100 units/ml penicillin and 100 μg/ml streptomycin, and incubated at 37°C in the presence of 5% CO_2_. P815 cells were seeded in a 96-well plate and treated with serially-diluted concentrations of the CTCM or saline for 24 hr. Cytotoxicity was measured using the 3-(4,5-Dimethylthiazol-2-yl)-2,5-diphenyltetrazolium bromide (MTT)-based *in vitro* Toxicology Assay Kit (Sigma-Aldrich, St. Louis, MO, USA) to determine the number of viable cells in proliferation. Cells were seeded at a density of 5000 cells/well in a 96-well culture plate. After cells were incubated with various concentrations of CTCM, the absorbance was recorded and analyzed according to the manufacturer's instructions. The cell numbers were determined using a standard curve constructed with known numbers of cells.

A total of 10^6^ cells/well of P815 cells were seeded in the wells of a 24-well cell culture plate. The mast cells were untreated or pretreated with the CTCM (100 μg/ml) or cromolyn sodium (a mast cell stabilizer, 10 μg/ml) for 20 min, and then incubated with Compound 48/80 (a mast cell activator, 10 μg/ml) for 9 hr. The cell culture media were collected and centrifuged at 150× *g* for 10 min at 4°C, and the supernatants were quantified in terms of the levels of histamine and IL-4 release, as described in the previous section.

### Statistical analysis

Results are expressed as the mean ± SEM. Repeated measures ANOVA with a Mauchly post-hoc test or One-way ANOVA with a Duncan post-hoc test were used for multiple-group comparisons. A *P*-value < 0.05 was taken to indicate significance.

## Results

### The CTCM has a bronchodilating effect

**Figure 1:**
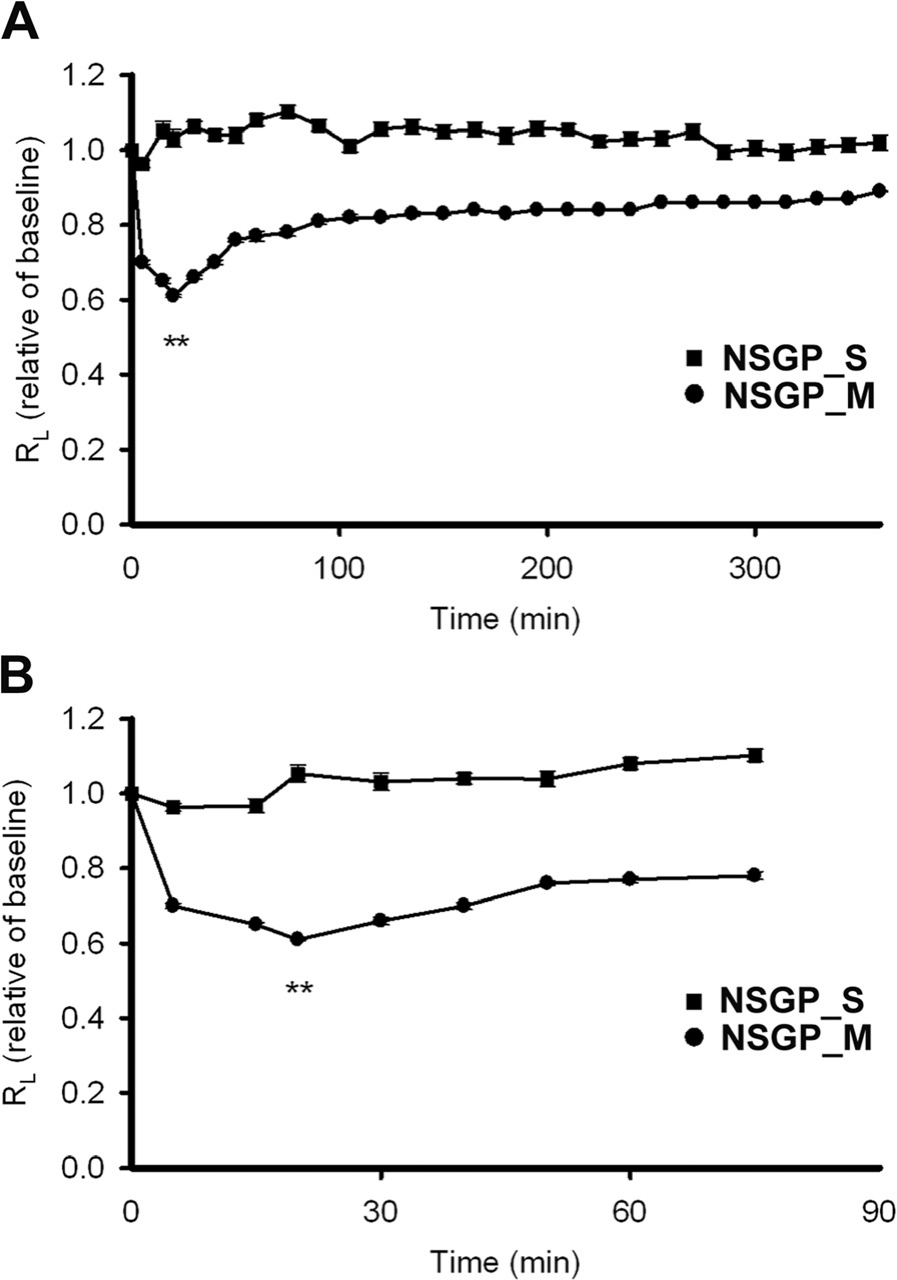
**The CTCM had a bronchodilator effect.** Guinea pigs were treated with a nebulized form of the CTCM, and the pulmonary resistance (R_L_) was significantly reduced (to 61.0 ± 0.01% of the baseline) 20 min post-CTCM treatment, while no change was seen in the animals treated with saline. **(A)** Overview of the 7-hr period. Comparison between the NSGP_S and NSGP_M groups. **(B)** Comparison of the different groups of sensitized animals in the first hour (repeated measures ANOVA with a Mauchly post-hoc test; **P <0.01). NSGP_S: unsensitized guinea pigs treated with saline by nebulization; NSGP_M: unsensitized guinea pigs treated with 0.06 g/ml CTCM for 5 min by nebulization.

First, we examined the effect of the CTCM on pulmonary resistance in the guinea pigs. In non-sensitized guinea pigs, those treated with the nebulized form of CTCM showed a significantly reduced pulmonary resistance as compared with the controls, and at 20 min post-CTCM treatment, the reduction reached a maximum (to 61.0 ± 0.01% of baseline, Figure 1). No change was seen in the animals treated with saline. This result indicated that the CTCM has a direct relaxant effect on the airway of the guinea pig.

### The bronchodilating effect of the CTCM is associated with the β-adrenoceptor

**Figure 2:**
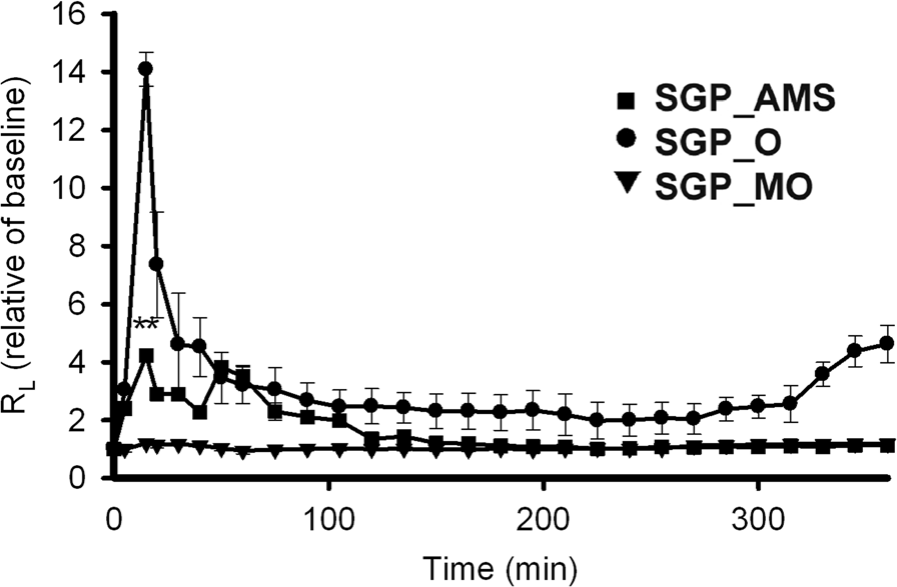
**The inhibitory effect of the CTCM on OVA-induced R**_**L **_**increase is mediated by the β-adrenergic receptor.** OVA-sensitized guinea pigs were pre-treated with the β-adrenoceptor antagonist propranolol, followed by CTCM inhalation or no CTCM inhalation before OVA challenge. Comparison between the SGP_O, SGP_MO and SGP_AMO groups of sensitized animals in a 7-hr period (repeated measures ANOVA with a Mauchly post-hoc test, SGP_AMO *vs*. SGP_MO; **P <0.01). Each point represents the mean of the data from 10-12 animals and the vertical bars represent the standard deviation. SGP_O: sensitized guinea pigs challenged with OVA by nebulization; SGP_MO: sensitized guinea pigs treated with 0.06 g/ml nebulized CTCM for 5 min, followed by OVA challenge by nebulization (at 5 min post-CTCM nebulization); SGP_AMO: sensitized guinea pigs pre-treated with β-adrenoceptor antagonist propranolol (1 mg/kg i.v.) for 3 min before treating with nebulized 0.06 g/ml CTCM for 5 min, and finally challenged by OVA by nebulization (at 5 min post-CTCM).

We previously showed that OVA challenge by nebulization caused the early antigen response of immediate bronchoconstriction, which peaked at 15 min in the sensitized guinea pigs (SGP) and increased the R_L_ by more than 13-fold that of the control group (unsensitized GP; NSGP) [28]. When CTCM treatment was administered to the guinea pigs 5 min before OVA challenge, the OVA-induced bronchoconstriction was almost completely impeded. In order to understand the mechanism underlying CTCM treatment, we investigated whether the β-adrenoceptor is involved in the bronchodilating effect of the CTCM. As shown in Figure 2, when OVA-sensitized guinea pigs were pre-treated with the β-adrenoceptor antagonist propranolol, the results showed that propranolol attenuated the inhibitory effect of the CTCM. The R_L_ in the SGP_AMO group was increased to approximately 3–4-fold that of the SGP_MO group (reaching a maximum at 15 min after challenge, *P* <0.01) in the first 30 min (Figure 2). Although the R_L_ of the SGP_AMO group was increased at 5 and 15 min and was maintained at a higher level similar to that of the SGP_O group, the R_L_ was decreased to a similar level to that of the SGP_MO group 2 hr after challenge. In addition, no late-phase responses were seen in the SGP_MO and SGP_AMO groups (Figure 2).

### The CTCM reduced the histamine and IL-4 levels in the BALF of OVA-challenged guinea pigs

**Figure 3:**
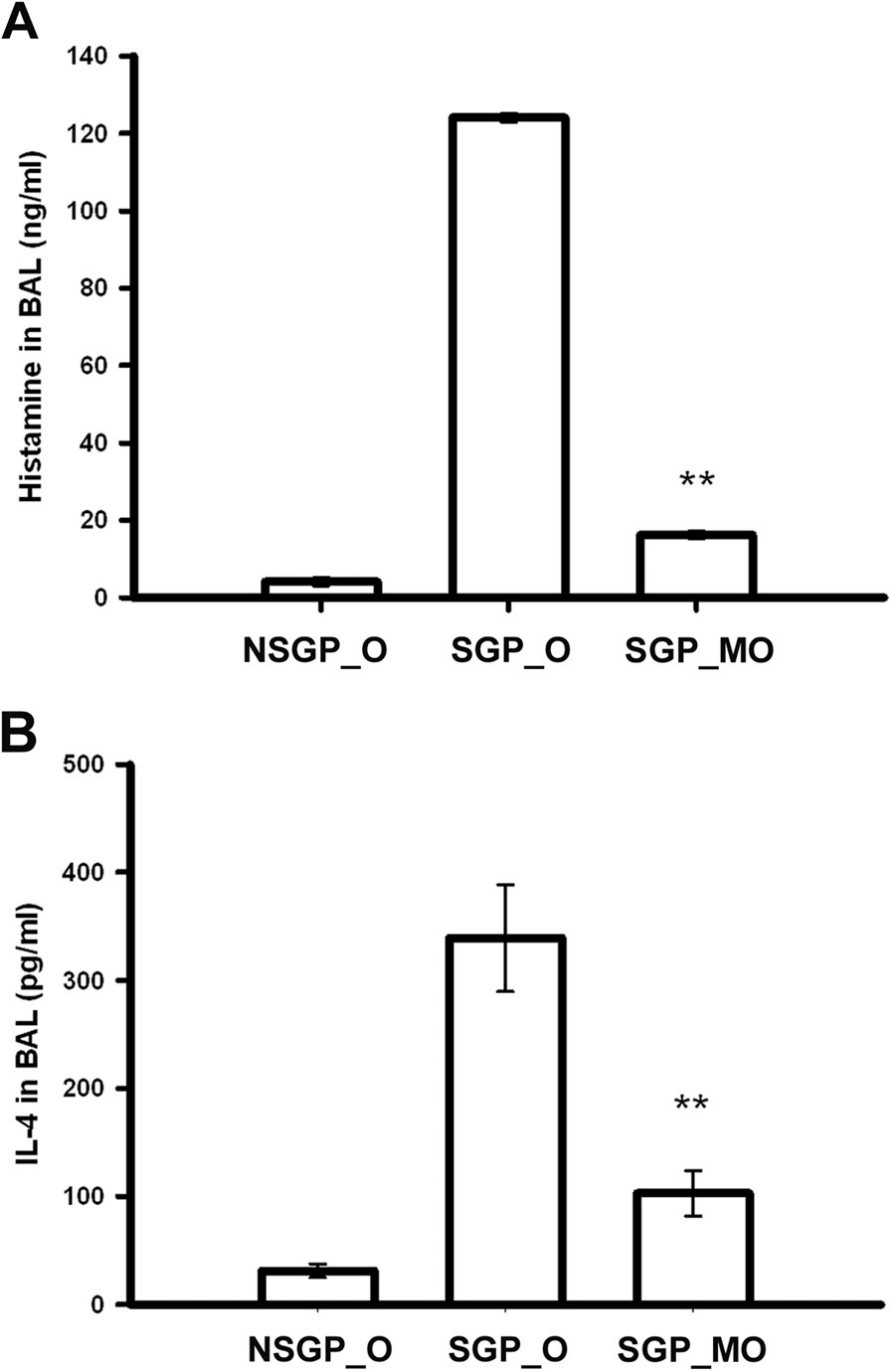
**Effect of the CTCM on the histamine and IL-4 levels in the BALF.** CTCM treatment before OVA challenge reduced the histamine and IL-4 levels in the BALF 5 min after OVA challenge (one-way ANOVA with a Duncan post-hoc test, **P <0.01: SGP_MO *vs.* SGP_O). Each group represents the mean of the data from 10 animals and the vertical bars represent the standard deviation. **(A)** Histamine and **(B)** IL-4. NSGP_O: unsensitized guinea pigs challenged with OVA by nebulization; SGP_O: sensitized guinea pigs challenged with OVA by nebulization; SGP_MO: sensitized guinea pigs treated with 0.06 g/ml nebulized CTCM for 5 min, followed by OVA challenge by nebulization (at 5 min post-CTCM nebulization).

At 5 min after OVA challenge, the histamine level in the BALF of the control NSGP group was only 4.11 ng/ml; this increased in the animals of the SGP_O group to 124.04 ng/ml. On the other hand, the histamine level in the group with CMCT treatment (SGP_MO) was only 16.23 ng/ml, which was close to that in the control group (NSGP_O) and significantly lower than that in the SGP_O group (Figure 3A). The IL-4 level in the BALF of the control group (NSGP_O) was only 30 pg/ml, and increased in the animals of the SGP_O group to 300 pg/ml. On the other hand, the IL-4 level in the group with CMCT treatment (SGP_O) was only 100 pg/ml, which was significantly lower than that in the SGP_O group (Figure 3B). The results suggested that mast cells, a major cell type in the airways, which secrete histamine and IL-4, may play a role in the OVA-induced airway hypersensitivity, and CTCM treatment might be able to stop the activation of mast cells.

### The CTCM reduced histamine and IL-4 release in activated P815 cells

**Figure 4:**
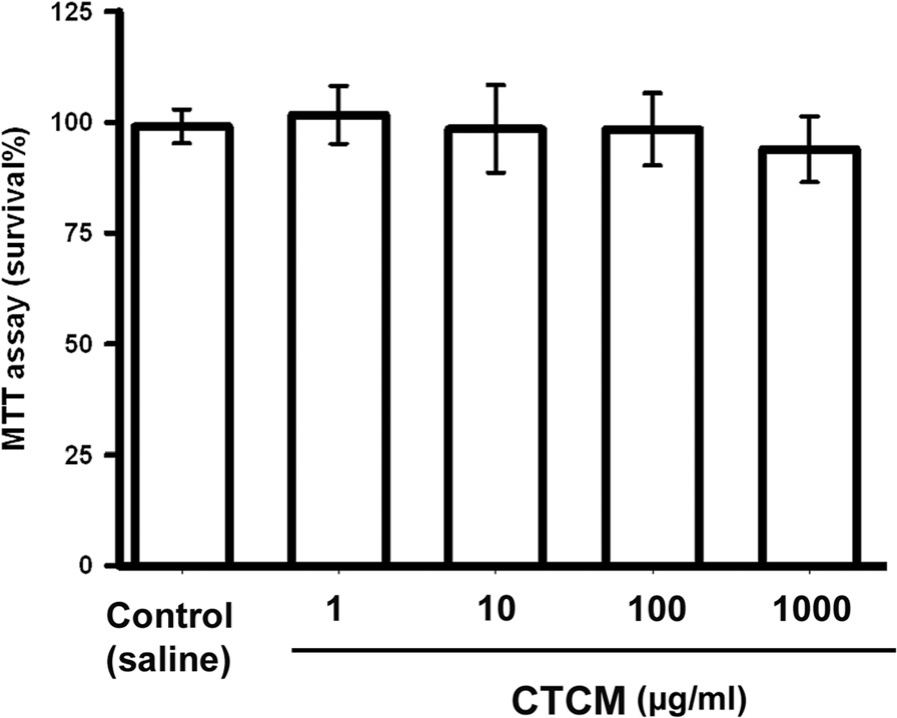
**Cytotoxic effect of the CTCM in P815 cells.** Various concentrations of CTCM (1, 10, 100 and 1000 μg/ml) were incubated with P815 mast cells for 9 hr. The cell viabilities were measured by MTT assay to determine the percentage of viable cells present. No significant difference between the CTCM-treated group and the control was seen (*P* >0.05).

In order to understand how CTCM treatment attenuates the airway hypersensitivity, we used an *in vitro* P815 cell culture to investigate the possible function of mast cells in this system. P815 cells were treated with the CTCM (1, 10, 100 and 1000 μg/ml) for 24 hr to evaluate the cytotoxicity of the CTCM towards the cells. MTT assay showed that P815 cells in the presence of even 1000 μg/ml of the CTCM did not exhibit significant cytotoxicity, and the cell viability was greater than 98% (Figure 4). The CTCM at the lower concentration of 100 μg/ml was then used for the subsequent experiment.

**Figure 5:**
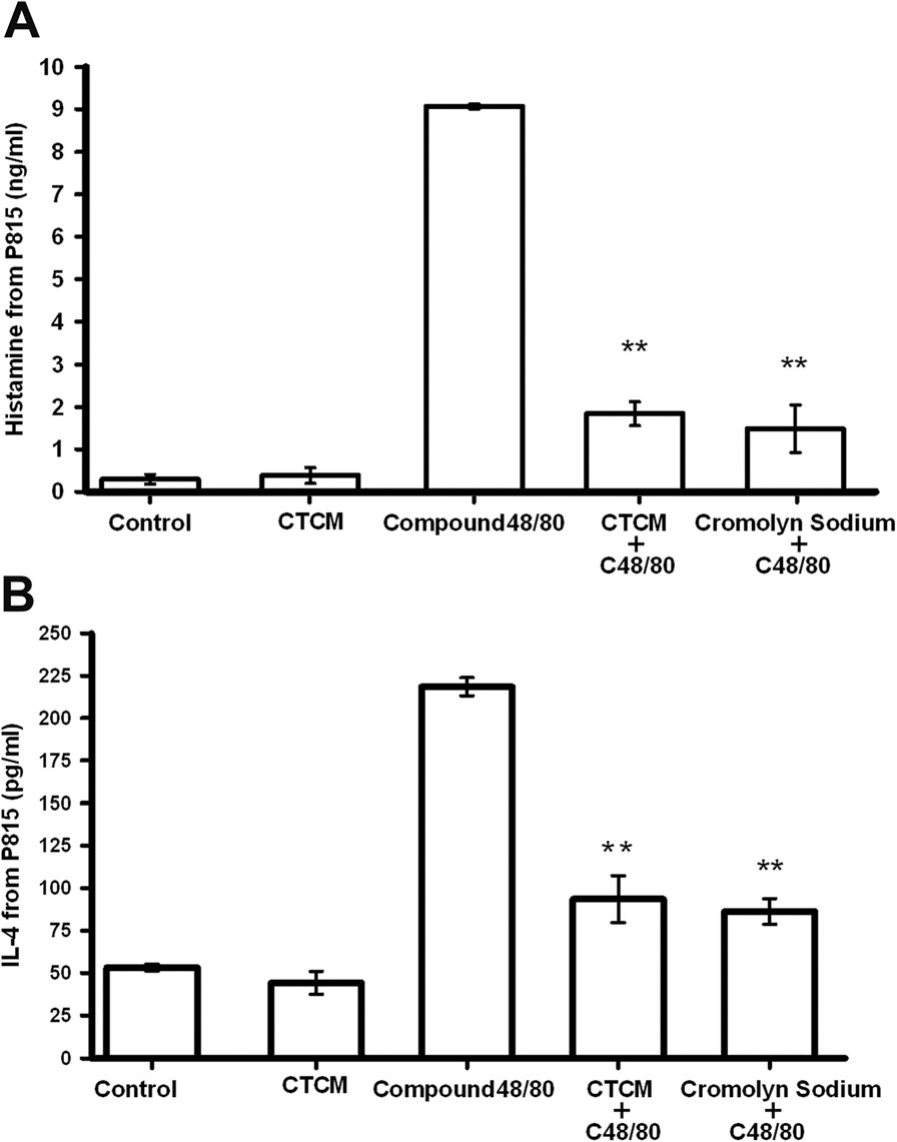
**Effect of the CTCM on the release of histamine and IL-4 in P815 mast cells.** Compound 48/80 was used to activate the P815 mast cells as a positive control, and cromolyn sodium was used to stabilize the mast cells as a negative control. The release of **(A)** histamine and **(B)** IL-4 in the culture medium with saline, “Compound 48/80 (C48/80) + CTCM (100 μg/ml)” or “Compound 48/80 + cromolyn sodium” were examined (one-way ANOVA with a Duncan post-hoc test **P <0.01; compared to Compound 48/80 treatment).

The baseline value for histamine release in the P815 cells was 0.29 ng/ml. No significant change was seen when the unactivated P815 cells were treated with CTCM alone. In the cells activated with Compound 48/80, the histamine release was increased to 9.06 ng/ml. Interestingly, when P815 cells were pretreated with the CTCM followed by Compound 48/80, the results showed a 5-fold decrease in histamine release. The inhibition ability of the CTCM was close to that of cromolyn sodium, a mast cell stabilizer (Figure 5A). On the other hand, Compound 48/80 treatment induced IL-4 release from a baseline of 53.10 pg/ml to 218.48 pg/ml (Figure 5B). Although the CTCM alone did not affect IL-4 release in the unactivated cells, CTCM attenuated the IL-4 level to 93.52 pg/ml in the Compound 48/80-activated mast cells, which was close to the inhibition effect (reduced to (86.24 pg/ml) of cromolyn sodium pre-treatment (Figure 5B).

**Figure 6:**
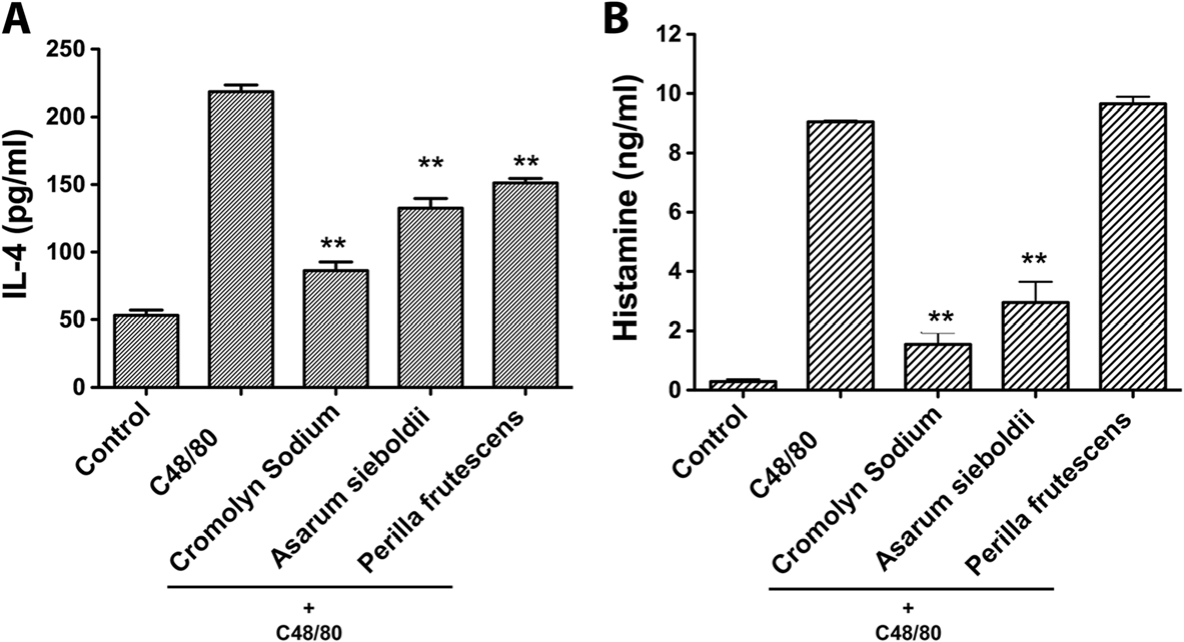
**Effects of individual herb treatment on the release of IL-4 and histamine in P815 mast cells.** Compound 48/80 (C48/80) was used to activate the P815 mast cells as a positive control, and cromolyn sodium was used to stabilize the mast cells as a negative control. The effect of “Individual herb + C48/80” or “cromolyn sodium + C48/80” on the release of **(A)** IL-4 and **(B)** histamine in P815 mast cells was examined. **P <0.01 (compared to Compound 48/80 treatment).

The effect of each individual herb was also tested. The results demonstrated that pre-treatment of cells with *Asarum sieboldii* resulted in a significant inhibitive effect on both IL-4 and histamine release, while *Perilla frutescens* treatment only had an inhibitive effect on IL-4 release (Figure 6). No effect was seen in the unactivated cells treated with the herbs, as well as the Compound 48/80-activated cells treated with other herbs (data not shown).

## Discussion

CTCMs have long been used in a wide range of diseases in many Asian countries for disease treatment and prevention. As the use of CTCMs is widely accepted by the people of these countries, the cost of treating diseases is covered by insurance in most of these countries. It is also gradually being considered by people in several European and American countries, especially its use in the treatment of chronic diseases owing to its effectiveness and the very minor side effects. The increase in the use of CTCMs and other herbal medicines emphasizes the need to understand the mechanisms of the medicines in order to develop safe and effective therapies.

The present study confirmed our previous findings that CTCMs administered in nebulized form delivered through the airways can inhibit OVA-induced airway hypersensitivity responses in guinea pigs [28], and further demonstrated that the effect of a CTCM against allergic bronchoconstriction acts partly through the β-adrenergic receptor, at least in the early airway response. Pre-treatment with propranolol intravenously 3 min before CTCM inhalation reduced the inhibition effect of CTCM in the animals during the first 90 min (Figure 2). No late airway response was seen after 5 hr of OVA challenge, which suggested that the CTCM had a long-lasting inhibitory effect on the antigen-induced airway hypersensitivity. On the other hand, our results demonstrated that the CTCM used in this study inhibited the increases in histamine and IL-4 levels in the BALF at 5 min post-OVA challenge. An *in vitro* experiment using P815 mouse mast cells revealed that the inhibition effect of the CTCM was similar to that of a mast cell stabilizer, cromolyn sodium. In addition, CTCM treatment had no effect on the basal histamine and IL-4 release in the unactivated mast cells. Our findings suggest that the CTCM directly inhibits the secretion of histamine and IL-4 from activated mast cells, and functions as a mast cell stabilizer. We observed that degranulation was reduced in the cells treated with CTCM; however, we have not further studied the underlying mechanism, and cannot conclude that the CTCM has the same targets as cromolyn sodium. Further studies will be required to identify the mechanism of the CTCM and investigate the benefits of the CTCM.

The reduction of IgE in BALF in our previous study [28] and decrease of histamine levels observed here suggest that the CTCM is effective in regulating both the early and late immune response.

Mast cells have been shown to play a key role in asthma through their release of a variety of mediators, including histamine, PGD2, tryptase, IL-4 and IL-5 [31]. The release of histamine from mast cells in the guinea pig is mediated by β-adrenoceptors [32], and β-adrenoceptor agonists have been shown to inhibit the anaphylactic release of mediators, including histamine, leukotriene C4, D4 and E4 and thromboxane A2, in an allergic sheep model [33]. Mast cell activation and the release of stored mediators and cytokines, including histamine and IL-4, are key events in asthmatic airways [31]. Histamine has been shown to account for over 50% of allergen-induced early asthmatic reactions [34,35]. The results of this study showed that the release of histamine was reduced after OVA challenge in sensitized guinea pigs pretreated with the CTCM. Our *in vitro* results further indicated that the CTCM inhibited the allergen-induced airway reactions through the stabilization of mast cells in the early-phase reaction after OVA challenge.

Several herbal medicines have been demonstrated to be effective in the treatment of asthma in animal models and humans, for example, antiasthma simplified herbal medicine intervention (ASHMI) [25], chai-po-tang (Japanese name: saiboku-to) [36,37], Ding-Chuan-Tang [21,38], *Houttuynia Cordata*[39], Guo-Min-Kang [26], Ma-Xing-Gan-Shi-Tang [23], San-Ao-Tang [24], and Xiao-Qing-Long-Tang [22] have all been shown to inhibit asthmatic responses effectively in animal models and/or in the treatment of patients. Studies have investigated the mechanisms of the therapeutic effect of herbal medicines in asthma; however, the mechanisms that explain the beneficial effect of many CTCMs remain unclear. In this study, we used nebulization and inhalation to administer the CTCM and investigated the underlying mechanism. Like bronchodilators, the CTCM can be inhaled after allergen attack, and the increased pulmonary resistance can be reduced. The inhibition of bronchoconstriction might occur through several pathways. First, the CTCM may stabilize mast cells and protect those cells from degranulation. Second, it may occur through the inhibition of released bronchoconstrictors (i.e., histamine). Third, the CTCM inhibits bronchoconstriction by blocking the receptors of those bronchoconstrictors, or the stimulation of β-adrenergic receptors relaxes the airway smooth muscle and reduces pulmonary resistance.

Ephedrae herba, a known source of ephedrine, has been used for the treatment of asthma for many years. A bronchodilatory effect of ephedrine mediated by its adrenomimetic property has been reported [40]. Although ephedrine has been reported to inhibit histamine and serotonin release from mast cells in a chemical-induced anaphylactic animal model [41], in our study no inhibition effect of Ephedrae herba was seen on Compound 48/80-activated P815 cells (data not shown). This could be due to the ephedrine level in the Ephedrae herba decoction being much lower than that in the study that directly used ephedrine. On the other hand, interestingly, our results also revealed that in P815 cells treated with *Asarum sieboldii* or *Perilla frutescens*, IL-4 production from the cells was attenuated, and *Asarum sieboldii* had the additional effect of reducing histamine release. No bronchoconstriction inhibition effect was seen when the animals inhaled the decoction of the rest of the individual herbs (data not shown). *Asarum sieboldii* is widely used in traditional medicine as it exerts anti-allergic [42] and anti-inflammatory [43] effects. Recent study has proved that several active compounds isolated from *Asarum sieboldii* have great anti-inflammatory abilities [44]. *Perilla frutescens* has also been reported to have a high antioxidant activity [45]. These two herbs may contribute majorly to reducing allergic tissue injury.

In our previous study [28], we demonstrated that the CTCM has an inhibitory effect on OVA-induced early and late asthmatic responses; in particular, the CTCM effectively reduced the serum IgE level, total infiltrated leukocytes, and IL-5 level in the BALF in the late-phase reaction to OVA challenge. Several studies have reported that eosinophils are important in the OVA-induced asthmatic response of animal models at 1 hr and later time points [22,46,47]. In the current study, we showed that mast cells are crucial in the early-phase reaction to OVA challenge, and CTCM treatment has the important function of stabilizing mast cells.

## Conclusions

In this study, we showed that the CTCM inhibited histamine and IL-4 secretion by stabilizing mast cells. Our findings suggested that the CTCM has the effect of a bronchodilator and exerts an anti-inflammatory effect in OVA-sensitized guinea pigs, and it can also stabilize mast cells in terms of the release of histamine and IL-4. This suggests that this CTCM can be effectively used for the treatment of asthma via inhalation.

## Competing interests

The authors declare that they have no competing interests to disclose.

## Authors’ contributions

HCC conceived and designed the study and carried out many of the culture experiments, analyzed and interpreted the data, and drafted the manuscript. CCG and CLC performed some of the experiments and data analysis, and contributed to the drafting of the manuscript. OTM was involved in the conception and design of the study and the supervision of experiments. All authors read the manuscript, contributed to its correction, and approved the final version.

## Pre-publication history

The pre-publication history for this paper can be accessed here:

http://www.biomedcentral.com/1472-6882/13/174/prepub

## Supplementary Material

Additional file 1: Figure S1 (A) Flow chart of the process of sensitization and challenge of the animals. (B) Diagram illustrates the method used to measure pleural pressure and lateral pressure for calculating the pulmonary resistance (R_L_). Click here for file
