# Supplementary figures and images for: A nebulized complex traditional Chinese medicine inhibits Histamine and IL-4 production by ovalbumin in guinea pigs and can stabilize mast cells in vitro
Source: BMC Complement Altern Med. 2013 Jul 13;13:174. doi: 10.1186/1472-6882-13-174 (PMC3716888; doi:10.1186/1472-6882-13-174)

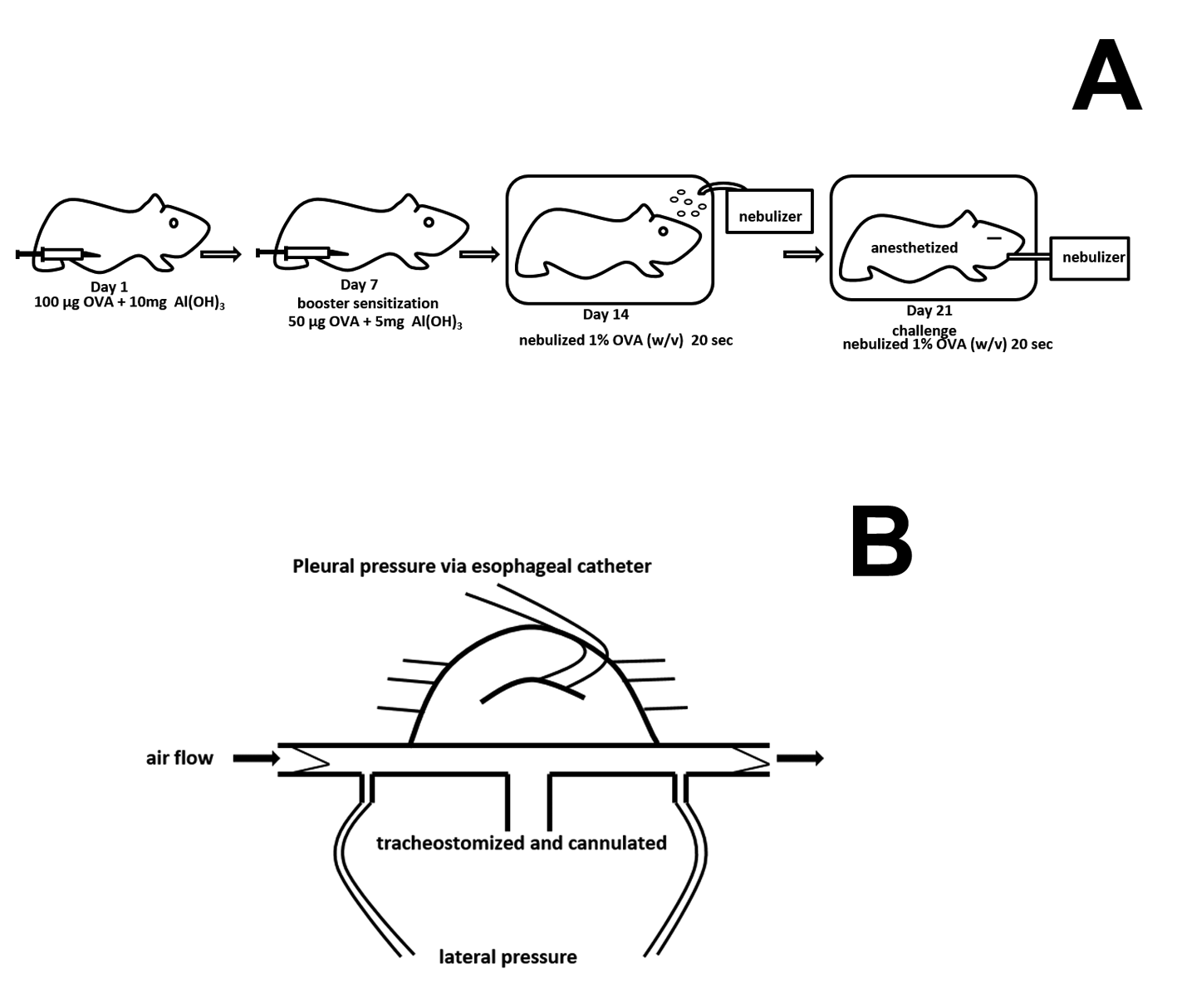

Supplement: Additional file 1: Figure S1 — (A) Flow chart of the process of sensitization and challenge of the animals. (B) Diagram illustrates the method used to measure pleural pressure and lateral pressure for calculating the pulmonary resistance (RL). [file 1472-6882-13-174-S1.tiff]
